# Role of AMPK in autophagy

**DOI:** 10.3389/fphys.2022.1015500

**Published:** 2022-11-25

**Authors:** Shengyuan Wang, Hongyan Li, Minghao Yuan, Haixia Fan, Zhiyou Cai

**Affiliations:** ^1^ Chongqing Medical University, Chongqing, China; ^2^ Department of Neurology, Chongqing General Hospital, University of Chinese Academy of Sciences, Chongqing, China; ^3^ Department of Neurology, Chongqing School, University of Chinese Academy of Sciences, Chongqing, China; ^4^ Chongqing Key Laboratory of Neurodegenerative Diseases, Chongqing, China; ^5^ Department of Neurology, The Affiliated Hospital of Southwest Medical University, Sichuan, China

**Keywords:** AMPK, autophagy autophagy, autophagosome autophagosome, lysosome, mTOR

## Abstract

Adenosine monophosphate-activated protein kinase (AMPK) is a significant energy sensor in the maintenance of cellular energy homeostasis. Autophagy is a highly conserved catabolic process that involves an intracellular degradation system in which cytoplasmic components, such as protein aggregates, organelles, and other macromolecules, are directed to the lysosome through the self-degradative process to maintain cellular homeostasis. Given the triggered autophagy process in various situations including the nutrient deficit, AMPK is potentially linked with different stages of autophagy. Above all, AMPK increases ULK1 activity by directly phosphorylating Ser467, Ser555, Thr574, and Ser637 at least four sites, which increases the recruitment of autophagy-relevant proteins (ATG proteins) to the membrane domains which affects autophagy at the initiation stage. Secondly, AMPK inhibits VPS34 complexes that do not contain pro-autophagic factors and are thus involved in isolation membrane forming processes, by direct phosphorylation of VPS34 on Thr163 and Ser165. After phosphorylation, AMPK can govern autophagosome formation through recruiting downstream autophagy-related proteins to the autophagosome formation site. Finally, the AMPK-SIRT1 signaling pathway can be activated by upregulating the transcription of autophagy-related genes, thereby enhancing autophagosome-lysosome fusion. This review provides an introduction to the role of AMPK in different stages of autophagy.

## Introduction

Adenosine monophosphate-activated protein kinase (AMPK), a serine/threonine kinase in eukaryotes, plays a crucial role in regulating cell energy balance in eukaryotes (Chen et al., 2018). When the ADP/ATP or AMP/ATP ratio is increased, AMPK is activated and triggers a series of physiological regulation processes (Mancini and Salt, 2018). Activation of AMPK increases the rate of catabolic (ATP-generating) pathways and compatibly decreases ATP usage. In addition, AMPK regulates cellular energy balance as well as whole-body energy metabolism (Liang et al., 2021; Wu et al., 2021). Dysregulation of energy balance plays an important role in the onset of many diseases, such as type 2 diabetes, obesity, and cancer (Hoffman et al., 2020). AMPK plays a central regulatory role in energy homeostasis, making it an important target for drugs to prevent and/or treat metabolic diseases (Suh et al., 2017).

Among eukaryotes, autophagy, a highly conserved and ubiquitous physiological process, is the intracellular catabolic pathway (de Pablos et al., 2019). Although autophagy is active to a certain extent at the physiological level, nutrient deficiency or energy deficiency is the main driving factor inducing autophagy in the physiological process (Wang et al., 2021). Autophagy is a lysosomal-mediated degradation system that has been highly conserved during evolution. Autophagy circulates cell contents and removes accumulated proteins, damaged organelles, and invading pathogens (such as bacteria and viruses) for the maintenance of normal cell function and intracellular homeostasis (Zhang et al., 2015; Newman et al., 2017). The process of autophagy is continuous, including sequential membrane reconstitution (Zhao et al., 2016; Miyamoto, 2019). This review describes the important role of AMPK in autophagy and metabolic regulation. We will discuss how AMPK activity is energy produced in the process of intracellular material synthesis and the catabolism pathway of macromolecules and explain how AMPK mediates signaling pathways by directly or indirectly regulating autophagy-related proteins and the activity of autophagy regulatory factors to explain the mechanism of AMPK regulation of autophagy.

## AMPK: Structures and activation mechanism

AMPK, a serine/threonine kinase, plays a crucial role in regulating cellular energy balance across eukaryotes. When the intracellular RATIO of AMP/ATP increases relative to ATP, AMPK is activated and targets a series of subsequent physiological processes (Ke et al., 2018). Activation of AMPK increases the rate of catabolic pathways, resulting in increased ATP production and a corresponding decrease in ATP use efficiency (Bonanno et al., 2019). AMPK also regulates whole-body energy metabolism in addition to maintaining intracellular energy balance (Seabright et al., 2020). AMPK plays a crucial role in the steady-state energy imbalance (Kishton Rigel et al., 2016), and the energy balance is one of the important inducing factors of many diseases, such as type 2 diabetes, obesity, and cancer, so the important role of AMPK in steady-state energy form to prevent and/or treat many metabolic diseases (including cancer) is an important drug target (Faubert et al., 2015).

**FIGURE 1:**
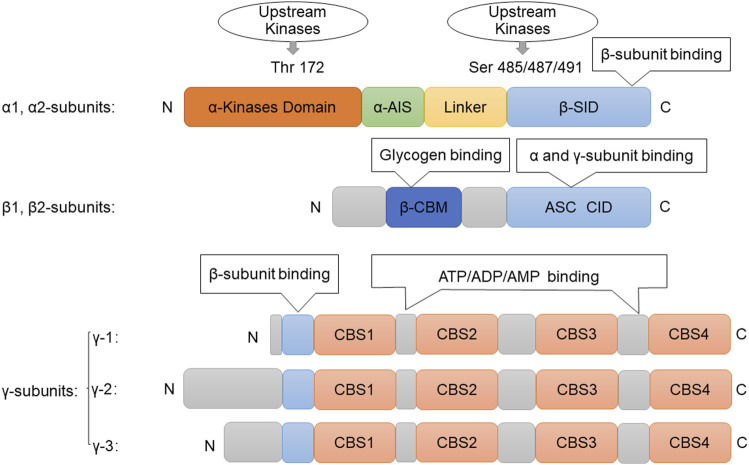
Structure of AMPK. Picture of the components of the AMPK subunits. The catalytic α-subunit can be phosphorylated at Thr172by upstream kinases including, LKB1, CaMKKβ, and TAK1, leading to enzyme activation. The β-subunit contains a glycogen-binding domain (GBD). The γ-subunits contain four nucleotide-binding modules (CBS domains), which can synergistically bind AMP, ADP, and ATP. Abbreviations: AMPK, adenosine monophosphate-activated protein kinase; ATP, Adenosine triphosphate; ADP, adenosine diphosphate; AMP, adenosine monophosphate; ASC, association with Snf1 complex domain; AIS, autoinhibitory sequence; CBM, carbohydrate-binding module; CBS, cystathionine-β-synthase domain; Thr, threonine; Ser, serine; GBD, glycogen bind domain; SID, subunit interacting domain.

AMPK is a highly dynamic trimer consisting of α, β and γ subunits, with the β subunit sandwiched between the α and γ subunits (Hardie et al., 2016). The α, β and γ subunits are subdivided into different subtypes: the α and β subunits contain two subtypes (α1, α2 and β1, β2), and the γ subunit can be divided into three subtypes (γ1, γ2 and γ3). Combinations of all subtypes are possible, resulting in 12 different AMPK complexes (Carling, 2017). The N-terminus of the α subunit contains the catalytic domain of serine/threonine-protein kinase and residues of Thr172, whose phosphorylation plays an important role in AMPK activation. The C-terminus of the α subunit contains domains necessary for interaction with the β and γ subunits. Amino acids 312–335 of the α subunit have domains responsible for autoinhibitory activity, and the β subunit is responsible for the interaction between glycogen and AMPK (Herzig and Shaw, 2018). The specific action of each subunit is due to its different properties, among which the N-terminus of the β subunit contains CBM (carbohydrate-binding module) dedicated to AMPK targeting glycogen. In addition, the C-terminal domain of the β subunit (amino acids 186–270) stabilizes the interaction between the α and γ subunits (Figure 1). In addition, the γ subunit contains four CBS (cythionine β-synthase) domains, which are involved in regulating the sulfur conversion pathway *in vivo* (Oligschlaeger et al., 2015). AMP, ADP, and ATP bind to the γ subunit of AMPK to catalyze structural changes in the α-subunit and regulate phosphorylation of upstream kinase and Thr172, thereby activating AMPK (Lin and Hardie, 2018; Lyons and Roche, 2018). Each binding site requires a tandem CBS pair to form a functional unit called the Bateman domain (CBS1+CBS2 = Bateman domain 1, CBS3+CBS4 = Bateman domain 2). There are three sites (CBS1, 3, 4) for binding AMP, ADP, and ATP-associated adenine nucleotides in the core structure of the mammalian AMPK complex. CBS1, 3, and four have their own functions. However, in CBS2, the Asp residues bound to ribosomes were replaced by Arg, while the nucleotide binding of CBS2 was not observed in the heterotrimer structure (Jeon, 2016).

AMPK is activated by binding of AMP through three distinct mechanisms in the canonical mechanism. Specifically, AMP first stimulates the phosphorylation of Thr172 by directly activating upstream kinases (Kim et al., 2020). Thr172 can be phosphorylated by several different upstream kinases, which establishes the main mechanism by which AMPK activity is regulated in the short term (Song et al., 2021). Different studies have suggested that LBK1 (liver kinase B1) kinase and MAPKKK family member TAK/MAP3K7 (transforming growth factor beta-activated kinase 1/mitogen-activated kinase 7) can respond to various signals by phosphorylating Thr172 (Fritzen et al., 2016). It has also been shown that Thr172 can be phosphorylated by CAMKK2 (calcium/calmodulin-dependent protein kinase 2) kinase in response to changes in calcium flux. Second, AMP can be allosterically regulated to make AMPK more attractive to its upstream kinase (Jang et al., 2018). Finally, AMP also protects Thr172 from phosphatase activity, leaving Thr172 phosphorylated and increasing AMPK activity (Hardie et al., 2012). AMPK can increase ATP levels in the body through two pathways. First, it enhances energy catabolic processes such as glucose metabolism, lipid oxidation, mitochondrial biogenesis, and autophagy. It can also be achieved by reducing energy anabolic processes, such as lipid synthesis, glycogen storage, gluconeogenesis, and protein synthesis.

## Autophagy biogenesis

Eukaryotes are highly conserved in their autophagy process, which is a degradation and recycling process (Ren and Zhang, 2018). It is possible that autophagy can create a cellular milieu that supports survival in some manner. The specific way is through the degradation of organelles, proteins and macromolecules to achieve the recycling of intracellular substances (Zhong et al., 2016). Depending on how the cytoplasmic material is transported into the lysosomal lumen, autophagy is classified into three main categories in mammalian cells: microautophagy, macroautophagy and chaperone-mediated autophagy (CMA) (Lamming and Bar-Peled, 2019). The three autophagy mechanisms are different (Cuomo et al., 2019). Macroautophagy occurs by fusion of autophagosomes and lysosomes, while microautophagy occurs by lysosomes directly wrapping and degrading the cell contents, while chaperone-mediated autophagy occurs by recognition of cytoplasmic proteins by molecular chaperones through special modules and then binding to special receptors on the lysosome membrane to enter the lysosome and be degraded (Mizushima and Komatsu, 2011; Martinez et al., 2016). All three types of autophagy can achieve lysosomal-mediated degradation and cycling of cytoplasmic components, including organelles, through different mechanisms (Yang and Klionsky, 2010; Lamming and Bar-Peled, 2019; Boukhalfa et al., 2021). Among them, macroautophagy is the most common type, and its biogenic process can be triggered by starvation, hypoxia, ER stress or other stimuli, thus involving a cascade of signaling and recruitment events (Corona and Jackson, 2018; Hu et al., 2021; Zeng and Jiang, 2021).

Once macrophages are activated, they specifically degrade damaged or excess organelles in the cell into metabolites for the biosynthesis process or energy generation in the cell so that other cells can survive and maintain the normal growth of the body (Parzych and Klionsky, 2014; Icard et al., 2021). Macroautophagy is generally protective for the body (Levine et al., 2011); however, excessive degradation may also cause damage to the body. Accordingly, the disruption of autophagy is associated with several human pathologies, such as lung, liver, and heart disease, neurodegeneration, myopathies, cancer, aging, and metabolic diseases (Kaur et al., 2021). Under the induction of the ULK1/2 complex, the nucleation of phagocytic groups initiates a series of subsequent biogenic processes of macroautophagy (Jayaraj et al., 2020; Karabiyik and Rubinsztein, 2021). The ATG12-ATG5-ATG16L1 complex, the class III PtdIns3K complex, LC3-II and ATG9 can assist phagocytic mass extension. Eventually, the cell component is enveloped by an expansive bilayer to form an autophagosome, and LC3-II divides from the outer membrane of the autophagosome (Nieto-Torres et al., 2021). Under certain conditions, autophagosomes fuse with endosomes to form amphisomes (Ho et al., 2017). In general, the outer membrane of autophagosomes fuses with lysosomes to form autophagolysosomes, and then the contents of autophagolysosomes are degraded and exported to the cytoplasm for cell reuse (Kimmelman and White, 2017).

## Regulation of autophagy by AMPK

AMPK plays a critical role in cellular energy metabolism and can act directly on the metabolism of proteins or indirectly affect the expression of genes related to the regulation of a variety of metabolic processes (Martin-Montalvo et al., 2013; Cordero et al., 2018). There are a number of downstream targets inhibited by AMPK, acetyl-CoA carboxylase (ACC), mammalian target of rapamycin (mTOR), fatty acid synthase (FAS), PPARγ coactivator 1α (PGC1α), and glycerol phosphate acyltransferase (GPAT), which are involved in fatty acid synthesis, protein synthesis, glycerolipid synthesis, and mitochondrial function. In this way, the energy balance is maintained, and the internal environment is kept in balance. Autophagy is a protective mechanism by which cells adapt to a variety of different types of stress, which is regulated by AMPK (Mihaylova and Shaw, 2011; Jia et al., 2020). By specifically phosphorylating autophagy-related protein complexes, AMPK promotes autophagy at multiple levels of autophagy regulation (Blagih et al., 2012).

**FIGURE 2:**
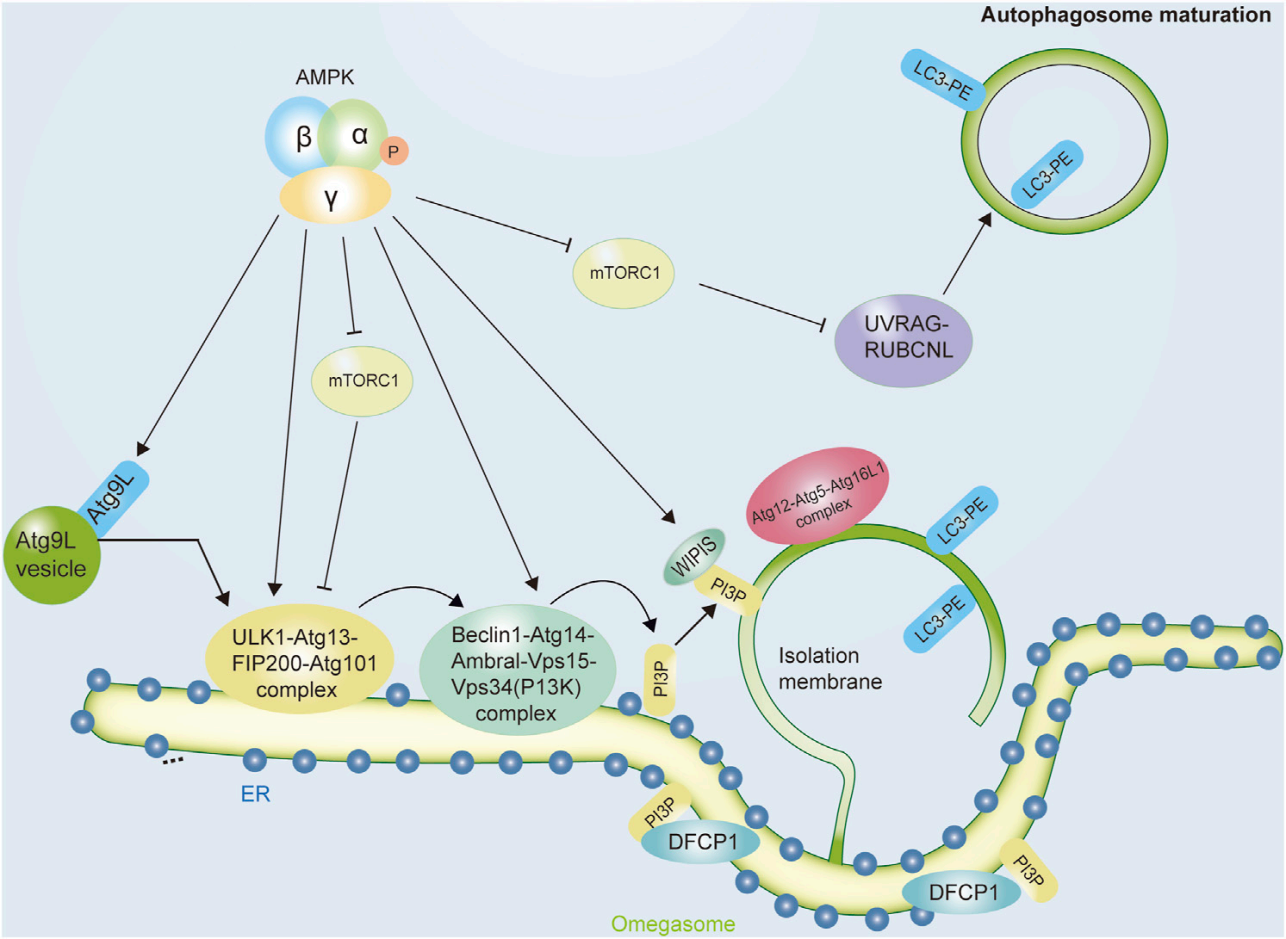
The role of AMPK in initiating the autophagic process. Autophagy, a lysosome-mediated degradation system, starts with the initiation and nucleation of a double membrane vesicle, known as the isolation membrane (or phagophore), in the cytoplasm. Autophagy occurs in a stepwise manner involving sequential membrane reshaping processes. Autophagy induction is initiated by the ULK1 complex, which contains ULK1, ATG13, FIP200, and ATG101. The activity of this protein complex is regulated by AMPK, which both inhibits the mTORC1 complex and activates the activity of ULK1 complexes. In mammalian cells, another complex ClassIII PI3K complex formed by VPS34, Beclin1, ATG14, AMBRA1, and other subunits act coordinately with the ULK1 complex to initiate autophagosome nucleation. AMPK can increase the pro-autophagic function of this complex and enhance its formation. Abbreviations: AMPK, adenosine monophosphate-activated protein kinase; ATG, autophagy-related protein; DFCP1, double FYVE-containing protein one; ER, endoplasmic reticulum; FIP200, focal adhesion kinase family interacting protein of 200kDa; LC3, microtubule-associated protein 1 A/1B-light chain three; PE, phosphatidylethanolamine; PI3P complex, the class III phosphatidylinositol (PtdIns) 3-kinase complex; mTOR, mechanistic target of rapamycin; mTORC1, mechanistic target of rapamycin complex one; ULK1, Unc-51-like kinase one; UVRAG, ultraviolet radiation resistance genes; WIPI proteins, WD-repeat protein interacting with phosphoinoside.

Autophagy is a lysosome/vacuole-dependent catabolic pathway in eukaryotes that is evolutionarily conserved (Lahiri et al., 2019). Nutrient deprivation or energy scarcity induces autophagy in a physiological manner (Yi et al., 2018). In organisms as diverse as yeast and humans, this intrinsic property remains unchanged. Thus, the involvement of AMPK in the regulation of this process seems logical (Tamargo-Gomez and MarinoAMPK, 2018). Autophagy begins with the formation of bowl-shaped membranes in the cytoplasm that expand into double-membrane vesicles called autophagosomes. Autophagosomes sequester cytoplasmic cargo and travel along the microtubule network until they merge with lysosomes (Mehrpour et al., 2010). In addition to the degradation of autophagosome cargo and its inner membrane, autophagosome-lysosome fusion also promotes the degradation of the autophagosome membrane. After degradation takes place, the resulting biomolecules are recycled back into the cytoplasm, where they are used to synthesize new biomolecules (Glick et al., 2010). ATG (autophagy-related proteins) are evolutionarily conserved genes/proteins that are necessary for the autophagy pathway. Originally described in yeast, these proteins are required for autophagosome formation, maturation, transport, or degradation, taking part in the various steps of autophagic degradation (Figure 2).

### AMPK facilitates the initiation of autophagy

During autophagy induction, ULK1 and VPS34 are the major components that drive autophagy initiation. The activation of AMPK induces autophagy *via* two different mechanisms: negative regulation of the mammalian target of rapamycin (mTOR) protein kinase complex and activation of ULK1 (Unc-51-Like Kinase 1, a mammalian ortholog of Atg1) by direct phosphorylation. ULK1 is a protein complex formed by interacting with ATG13, RB1CC1/FIP200, and ATG101. Even though there are many signaling pathways that can influence autophagy, two of the most important ones are AMPK and mTOR (mechanistic/mammalian target of rapamycin). AMPK positively regulates autophagic activity. At least four sites on ULK1 are phosphorylated by AMPK to stimulate its activity. To regulate the activity of the ULK complex, AMPK antagonizes mTORC1 (Kim et al., 2015; Park et al., 2016). There are two protein complexes formed by mTOR, mTORC1 and mTORC2 (Egan et al., 2011; Gonzalez et al., 2020). Autophagy regulation is insignificantly influenced by mTORC2 (Schmitz et al., 2021). In contrast, a mammalian cell’s main autophagy suppressor is mTORC1 (Schmitz et al., 2021). As mTORC1 is normally activated when energy levels are high, cellular amino acid content is high, or growth factors are stimulated, these conditions are damaging to autophagy (Cohen and Hall, 2009). Due to their antagonistic roles, it is known that AMPK increases autophagic degradation by inhibiting mTORC1 activity (Rabinowitz and White, 2010), and the activity of mTORC1 is a molecular pathway that is closely related to that of AMPK (Tamargo-Gomez and MarinoAMPK, 2018; Yoo and Jung, 2018). When sufficient energy/growth factors or amino acids were present, mTORC1 inhibited autophagy by inhibiting phosphorylation of ATG13, thereby reducing the activity of the ULK1 complex and thus the rate of autophagosome formation. Similarly, ULK1 itself is a direct target of mTORC1, so mTORC1 inhibits the autophagic process by targeting ULK1 and ATG13 (Kim et al., 2011; Huang et al., 2020).

According to AMPK, a variety of pro-autophagic stimuli negatively regulates ULK1 complex activity, whereas mTORC1 positively regulates ULK1 complex activity (Zhao and Zhang, 2016). AMPK increases ULK1 activity by directly phosphorylating Ser555, Thr574, Ser467, and Ser637. The formation of autophagosomes is enhanced by increasing the recruitment of autophagy-relevant proteins to membrane domains. Furthermore, AMPK inhibits ULK1 inhibition by both inhibiting mTORC1 and blocking its inhibitory activity. First, in addition to activating TSC2(Tuberous sclerosis complex 2) by phosphorylating its Thr1227 and Ser1345 residues, AMPK also negatively influences the activity of mTORC1 by promoting the assembly of the heterodimer between TSC1 and TSC2 (Huang and Manning, 2008; Egan et al., 2011; Pengo et al., 2017). Second, inhibition of mTORC1 by AMPK is brought about by direct phosphorylation of the Ser722 and Ser792 residues in RAPTOR (Regulatory-associated protein of mTOR) (Johnson et al., 2013).

AMPK regulates the activity of Class III PI3K complexes as well (Kahn et al., 2005). It is also through phosphorylation in different Class III PI3K subunits that AMPK regulates autophagy, it also modulates their affinity for other complex components, including VPS34 itself (Kim et al., 2013a). Therefore, AMPK controls the relative abundance of various VPS34-containing complexes, which in turn regulates cellular energy levels through vesicle trafficking (Yang et al., 2011). Furthermore, divergent regulation of Vps34 complexes by AMPK occurs in the context of nutrient stress and autophagy (Kim et al., 2013a; Yoon et al., 2016). Studies have shown that AMPK regulates the balance of Class III PI3K complexes in this regard (Kim et al., 2013b). Beclin1’s binding to VPS34 and ATG14 is increased when AMPK phosphorylates Thr388 in Beclin1, the higher autophagy activity is promoted upon glucose withdrawal than in a wild-type cell (Zhao et al., 2015). A similar effect can be seen when AMPK phosphorylates mouse Beclin1 at Ser-91 and Ser-94 under nutritional stress (Stjepanovic et al., 2017).

Additionally, it interacts with different Class III PI3K complex components, AMPK can influence their composition by phosphorylating other proteins that are essential for the formation and stability of VPS34-containing complexes (Russell et al., 2013). It has also been demonstrated that AMPK-mediated phosphorylation of an ATG14 L/VPS34 scaffolding protein, Thr32 on PAQR3 (progestin and adipo-Q receptors member 3), or that of Thr50 on the VPS34 associated protein RACK1 (Receptor for activated C kinase 1) enhances stability and pro-autophagic activity of Class III PI3K complexes (Russell et al., 2013; Park et al., 2016). In parallel with the activation of VPS34, it is also phosphorylated in a variety of components of the pro-autophagic complexes, AMPK, which is generally pro-autophagic, suppresses the activity of late autophagy PI3KC3–C2 by phosphorylating Thr163 and Ser165 in VPS34. Therefore, in conditions that promote autophagy, by activating AMPK, pro-autophagic VPS34 complexes are activated while other Class III PI3K complexes involved in autophagy-independent processes are inhibited (Xu et al., 2016).

### AMPK promotes autophagosome biogenesis

Phagophore expansion is thought to be responsible for autophagosome formation; that is, in autophagosomes, a sheet of membrane equal to the size of a complete autophagosome does not separate from the endomembrane system, but simply curls up (Grasso et al., 2018). The formation of autophagosomes is thought to be influenced by almost every compartment of the membrane, according to numerous lines of evidence, counting the mitochondria, ER, plasma membrane, and Golgi apparatus (Kimura et al., 2007). It appears that autophagosome generation may be mediated by at least two mechanisms (Yang and Klionsky, 2010; Kageyama et al., 2021). In yeast, membranes are believed to be delivered from various organelles, and Another system uses an omega-shaped membrane structure, called an omegasome, which is derived from phosphatidylinositol-3-phosphate (PtdIns3P)-enriched ER subdomains. Autophagosomes of yeast are formed from a single preautophagosomal structure (PAS), where most Atg proteins are recruited (Xie and Klionsky, 2007). There is a hierarchy of recruitment of the Atg protein to the PAS in yeast based on genetic studies (Polson et al., 2010). Upstream of Atg1 are the Vps34 lipid kinase complex and the Atg1 protein kinase complex (Reggiori and Klionsky, 2005). PAS expansion into a phagophore and autophagosome closure are dependent on Atg proteins downstream of these initiating complexes (Galluzzi and Green, 2019).

On the phagophore surface, Atg12 and Atg5 are covalently linked, triggering autophagosome formation (Nishimura et al., 2017). Atg16 and a second cytosolic Atg12-Atg5 conjugate are drawn to this compartment, where they begin to redistribute and focus primarily on the outside lipid bilayer. As Atg12-Atg5-Atg16 form larger oligomers, they determine phagophore elongation and curvature through the delivery of Atg8-containing membranes. After the autophagosome has been formed, Atg12 and Atg16 dissolve from this structure, while Atg4 releases the lipid bilayer-bound Atg8 to the cytosol by proteolytically cleaving it (Kawabata and Yoshimori, 2020). When the coating is uncoated, the autophagosome fuses with the lysosome. AMPK-MTORC1-ULK1/2 signaling axis regulates autophagosome formation in response to amino acid availability and energy balance in cells. It has been suggested that AMPK plays a role in the maturation of autophagosomes. Through UVRAG phosphorylation, AMPK can inhibit mTORC1 signaling, suppressing autophagosome maturation (Dunlop and Tee, 2013).

### AMPK regulates autophagosome-lysosome fusion

The next stage of autophagy is the fusion of the outer membrane of autophagosomes and lysosomes to form autolysosomes and finally transport the materials encapsulated in autophagosomes to the lysosomal lumen (Mauthe et al., 2018). Autolysosomes degrade the inner membrane of the autophagosome and its encapsulated substances (Zhou et al., 2013). Moreover, the formation of autophagosomes requires a specific period. Studies have shown that only closed autophagosomes can fuse with lysosomes to form autolysosomes. However, how is the closure of autophagosomes regulated? In mammals, a defect in the ATG-conjugation system results in the accumulation of unclosed autophagosomes, implying that it is likely to function in the elongation and closure of autophagosomes and is important for the transition of the isolation membrane into the autophagosome (Nieto-Torres et al., 2021). It also participates in the maturation of autophagosomes, it was shown that the ATG conjugation system (consisting of ATG3, ATG7, and ATG5) is essential for degrading inner autophagic membranes; however, it is not required for autophagosome-lysosome fusion. In contrast, according to another study, cell lines that were knocked out of the entire ATG8 protein family showed that Although LC3 and GABARAP proteins do not participate in autophagosome formation, they play an essential role during lysosome-autophagosome fusion (Itakura et al., 2012).

Late endosomes and lysosomes are predominantly found in the perinuclear region, while autophagosomes form at random in the cytoplasm (Savini et al., 2019). Thus, once autophagosomes have been produced, they must be delivered to the perinuclear region. Dynein-dynactin motor complexes move cargo to the perinuclear region, while most kinesins drive cargo toward the periphery of the cell with their plus-end directed motor proteins (Yamamoto and Noda, 2020). Autophagosomes are transported minus-end-directed, given that lysosomes are found in perinuclear regions, and indeed, live imaging reveals mature autophagosomes traveling along microtubule tracks to lysosomes. By microinjecting antibodies against LC3, autophagosome movement is inhibited, suggesting LC3 plays a role during autophagosome movement. Furthermore, Autophagosome positioning requires plus-end-directed transport mediated by kinesin because the depletion of KIF5B blocks autophagy and causes clustering of autophagosomes in the perinuclear space (Lorincz and Juhasz, 2020). It is interesting to note that lysosome localization affects the rate of autophagosome fusion. Thus, it is crucial for fusion to coordinate autophagosome and lysosome transport. However, how do autophagosomes and lysosomes connect with microtubules? Rab7 is a small GTPase that acts as a molecular switch and, presumably, Connects autophagosomes and microtubule motors through FYCO1(FYVE and coiled-coil domain-containing 1) in late autophagosomes, thereby kinesin-driven movement towards the cell periphery is mediated (Nakamura and Yoshimori, 2017). The Rab7 protein also functions in the reverse direction by facilitating the transport of autophagosomes, autolysosomes, and lysosomes to the perinuclear region through its interaction with Rab-interacting lysosomal protein (RILP), dynein and the cholesterol sensor ORP1 L (also known as OSBPL1A) (Feng et al., 2013). Actin filaments utilize the myosin family of motor proteins for moving intracellular charges similarly to microtubules (Zhao et al., 2021).

Autophagosome fused with the endocytic system is the result of its arrival at its destination. However, since both processes seem to occur almost simultaneously, it is difficult to distinguish movement from fusion. Currently, In order to understand this process, we need to understand intracellular membrane trafficking in general, a particular focus is on three protein families: membrane-tethering complexes, Rab GTPases, and soluble N-ethylmaleimide-sensitive factor attachment protein receptors (SNAREs) (Tian et al., 2020). As Rab proteins localize to specific membranes, they recruit tethering complexes that act as bridges between compartments intended for fusion. In turn, these tethering complexes enable SNARE proteins to drive the fusion of opposing lipid bilayers. By binding to LC3 and STX17–SNAP29, EPG5 binds to late endosomes/lysosomes along with Rab7 and VAMP-8; In this way, the trans-SNARE complex can be assembled for fusion more easily (Wang et al., 2016). Unlike EPG5, ATG14 L binds to STX17 and STX17-SNAP29 SNARE complexes on autophagosomes, but not to STX17–SNAP29-V AMP8 RQabc SNARE complexes, suggesting that ATG14 L appears to act earlier than EPG5. An adaptor protein, PLEKHM1, interacts with Rab7, LC3 proteins, HOPS–SNARE complexes, and GABARAP proteins to facilitate fusion between autophagosomes and lysosomes. It is still unclear how EPG5, ATG14 L, and the HOPS complex function as tethering factors. The O-GlcNAcylated SNAP-29, which is generated by O-linked N-acetylglucosamine transferase (OGT), has a reduced affinity for its SNARE partners. By decreasing levels of O-GlcNAcylated SNAP-29, starvation suppresses this modification, and SNAP-29-containing trans-SNARE complexes are assembled, stimulating autophagy.

**FIGURE 3:**
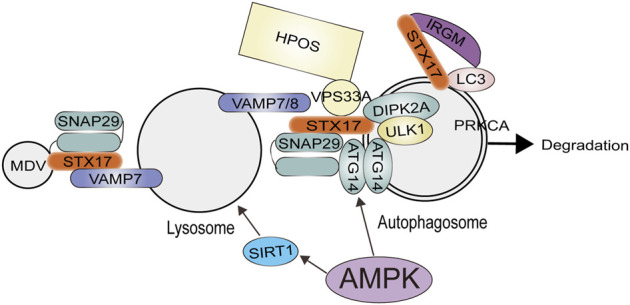
The role of AMPK for Autophagosome-lysosome fusion of autophagosomes with lysosomes involves the concerted action of RABs, tethers, and the SNARE complex. AMPK can positively regulate lysosomal biogenesis and function. AMPK activates ATG14 thereby promoting the fusion of the autophagosome and lysosome. Abbreviations: ATG14, autophagy-related protein14; DIPK2A, divergent protein kinase domain 2A; HOPS, homotypic fusion and protein sorting; IRGM, immunity related GTPase M; MDV, mitochondrial-derived vesicle; PRKCA, Protein Kinase C Alpha; SNAP29, synaptosomal associated protein 29; STX17, syntaxin 17; VAMP7, vesicle associated membrane protein seven; VPS33A, vacuolar protein sorting 33 homolog A; ULK1, unc-51 like.

There is robust evidence that AMPK plays a crucial role in autophagosome-lysosome fusion (Figure 3), and the activation of AMPK can positively regulate lysosomal biogenesis and function (Carroll and Dunlop, 2017). In mammals, some studies have shown that the coactivator-associated arginine methyltransferase 1 (CARM1) plays a crucial role in autophagy. A genome-wide analysis reveals that CARM1 acts as a transcriptional coactivator by interacting with transcription factor EB (TFEB) (Huang et al., 2019; Fang et al., 2021). An AMPK–SKP2–CARM1 signaling axis is responsible for regulating autophagy induction after nutrient starvation by CARM1-dependent histone arginine methylation, a crucial nuclear event in autophagy. Under nutrient-rich conditions, CARM1 stability is regulated by the SKP2-containing SCF (SKP1-cullin1-F-box protein) E3 ubiquitin ligase in the nucleus, but not in the cytoplasm (Shin et al., 2016). In addition, this study suggests that nutrient deprivation causes nucleus FOXO3a to undergo AMPK-dependent phosphorylation, which in turn inhibits transcription of the SKP2 gene. CARM1 is upregulated as a result of this repression, and it acts as a coactivator of TFEB in regulating lysosome function. As a result of glucose starvation, a lysosomal AMPK-activating complex is formed composed of AMPK and, V-ATPase, liver kinase B1 (LKB1), aldolase, AXIN, and Ragulator-RAG (Holland et al., 2020). AMPK is effectively activated by lysosomal N-myristoylation and DNA-dependent protein kinase (DNA-PK)-mediated phosphorylation of its γ-subunit. In contrast to mTORC1, AMPK inhibits diverse anabolic pathways while promoting lipolysis, glycolysis, and macroautophagy. Growth factors and cellular energy status are also involved in microtubule-mediated lysosomal trafficking. AMPK is activated by resveratrol when it interacts with its direct target SIRT1. Resveratrol has been shown to protect against Parkinson’s disease by enhancing autophagy in various models (Moors et al., 2017). It is a well known phenomenon that exercise increased the brain’s lysosome and autophagy levels. Exercise also promotes the nuclear translocation of TFEB in the cortex, increasing the transcription of genes related to autophagy and lysosomes. Fang Lin’s team discovered that exercise activated autolysosomal function and the gene transcription regulated by TFEB in the cerebral cortex through AMPK‐SIRT1‐TFEB pathway (Huang et al., 2019). Moreover, it has been shown that lysosomal function is activated by AMPK inhibition of mTORC1, and mTORC1 localization to lysosomes is not directly related to its role in regulating lysosomal function. In addition, as a result of mTORC1 suppression, TFEB activation is necessary, but not sufficient to activate lysosomes (Carroll and Dunlop, 2017). The expression of autophagy and lysosome genes is repressed by BRD4 in a study by Sakamaki et al. This inhibition is mitigated by AMPK-SIRT1 signaling during nutrient deficiency, thus allowing autophagy activation. By blocking BRD4 function, autophagy and lysosomal function are enhanced, as well as protein aggregate degradation is promoted (Sakamaki et al., 2017).

## Conclusion and perspectives

In higher eukaryotes, the regulation of autophagy has become increasingly complex, and multiple signal cascades are related to key autophagy factors. However, AMPK may still be the main molecular autophagy inducer that counteracts the activity of mTORC1, and mTORC1 has also been the main molecular autophagy inhibitor during the evolutionary process. AMPK inhibits anabolic activities that are activated due to a lack of energy or nutrients and switches off cell growth. While regulating the function of autophagy, AMPK’s role in adapting cell metabolism to energy supply is also conserved in various organisms from yeast to mammals.

As discussed in this review, AMPK plays a key role in the regulation of autophagy, coupled with various processes for which autophagic activity is beneficial, suggesting a potential mechanistic involvement of autophagy for some of the positive effects of AMPK activation. Future studies aimed at dissecting the precise molecular mechanisms by which AMPK exerts its wide variety of beneficial effects on human health will shed more light on these questions.
